# Editorial: Secretomics: More secrets to unravel on plant-fungus interactions, volume II

**DOI:** 10.3389/fpls.2023.1123403

**Published:** 2023-01-13

**Authors:** Delphine Vincent, Dominique Job, Jan Schirawski, Martijn Rep, Maryam Rafiqi

**Affiliations:** ^1^ Agriculture Victoria Research, AgriBio, Bundoora, VIC, Australia; ^2^ Centre National de la Recherche Scientifique (CNRS)/Université Claude Bernard Lyon 1/Institut National des Sciences Appliquées/Bayer CropScience Joint Laboratory (UMR 5240), Bayer CropScience, Lyon, France; ^3^ Matthias-Schleiden-Institute/Genetics, Friedrich Schiller University Jena, Jena, Germany; ^4^ Swammerdam Institute for Life Sciences, University of Amsterdam, Amsterdam, Netherlands; ^5^ AgroBioSciences Program, Mohammed VI Polytechnic University (UM6P), Ben Guerir, Morocco

**Keywords:** secretome, glycoside hydrolase, *Fusarium oxysporum*, *Botrytis squamosa*, *Botrytis elliptica*, biometric pipeline

Understanding the mechanisms underlying plant-fungal interactions is paramount to develop new disease control strategies. The association between plants and fungi is a two-way process, yielding alterations in both partners largely initiated and sustained *via* secreted components. The sum of the secreted compounds released into the extracellular space is called the secretome. Secretomics, the comprehensive study of molecules that are secreted by a cell, a tissue or an organism, has become a key methodological approach to unlock the secrets of plant-microbe interactions.

## Secreted glycoside hydrolases

Fungal and oomycete microbial glycoside hydrolases (GHs) delivered to cell surfaces and surrounding extracellular environments during host colonisation were reviewed by Bradley et al. The microbial nutritional lifestyle is associated with the number and type of GHs secreted. Many secreted microbial GHs promote plant colonisation and as such, serve as virulence factors with a myriad of functions, including nutrient acquisition, detoxification and manipulation of plant microbiota. Interestingly, these enzyme effectors can either suppress plant immune responses by preventing activation of microbe recognition by plant pathogen receptors (PPRs), or conversely activate host immunity as microbe-associated molecular patterns (MAMPs) or by creating damage-associated molecular patterns (DAMPs) as a consequence of GHs’ enzymatic activity. This review draws a comprehensive picture of secreted GHs and their critical roles in plant infection by fungi and oomycetes. In addition to providing thorough insights into this layer of plant-pathogen interaction, current knowledge gaps that should be addressed by future research are identified.

## Programmed cell death-inducing proteins of Botrytis species

Necrotrophs have evolved specialized proteins that actively induce plant cell death by co-opting the programmed cell death (PCD) machinery of the host, in order to access nutrients. *Botrytis squamosa* and *Botrytis elliptica* are necrotrophic fungal pathogens of onion and lily, respectively. Both fungi are closely related and assumed to share a considerable number of proteins to induce host PCD. This hypothesis was confirmed by Malvestiti et al. using genomics to disclose a high level of synteny between the two species, with a few balanced structural chromosomal arrangements. Post-genomics (secretomics and transcriptomics) analyses of culture filtrates inducing cell death responses upon leaf infiltration identified orthologous cell death-inducing proteins displaying similar expression patterns during infection of their respective host. Such comparative -omics studies are greatly needed to shed light on the mechanisms that make some pathogens more host-specific than others.

## The root fungal pathogen *Fusarium oxysporum*

The root fungal pathogen *Fusarium oxysporum* causes vascular wilt diseases of economically important crops throughout the world and, as a result, is actively researched in breeding programs that target crop resilience. Based on their host specificity, *F. oxysporum* isolates are grouped into *formae speciales*. Because of the genetic heterogeneity and polyphyletic nature of *F. oxysporum* strains, their assignment to *formae speciales* using non-experimental procedures remains challenging. To address this, Brenes Guallar et al. have developed the *Fusarium oxysporum* Effector Clustering (FoEC) pipeline that processes genome assemblies to classify strains by *forma specialis* based on hierarchical clustering of the presence of predicted putative effector sequences. The updated FoEC2 pipeline was improved to be more user-friendly, customizable and to offer greater multithreading scalability. FoEC2 was applied to 537 *F. oxysporum* genomes to successfully classify isolates into *formae speciales* and identify their subtypes. The FoEC2 pipeline allows reducing the workload and number of experiments required to confirm host specificity and presents a valuable tool to minimise the time and maximise the resources needed to characterize newly assembled genomes.

*F. oxysporum* secretes numerous effectors into the extracellular space, including Foa3 that is internalised by plant cells and suppresses molecular pattern-triggered defense responses. Tintor et al. performed a functional characterisation of this effector. Foa3 suppresses defense responses irrespective of whether the protein carries a secretory signal peptide or not, suggesting that Foa3 harbours an internalisation signal. Foa3 localizes to mobile subcellular structures of unknown identity, among other subcellular localizations. Foa3 can deliver an orthotospovirus avirulence protein-derived peptide into the cytosol, activating the matching resistance protein. Foa3 can also cause a strong suppression of pattern-triggered immune responses even in absence of the pathogen. This paper is an important contribution to the mystery of how fungal effectors translocate into host plant cells, a very timely research field that is critical to understand the full picture of effector biology and molecular and cellular plant-microbe interactions.

In tomato-infecting *F. oxysporum* strains, the genes for the effectors Six3 (Avr2) and Six5 form a gene pair on the pathogenicity chromosome. Whilst it was known that Avr2 suppresses plant defence responses and is required for full pathogenicity, Six5’s role warranted further investigation. Using transgenic Arabidopsis lines expressing *SIX5*, Blekemolen et al. report that the effectors Avr2 and Six5 function together to expand the size exclusion limit of plasmodesmata *via* un unknown mechanism to enable cell-to-cell movement of the effectors Avr2, Six6 and Six8. The unique combined functions of the Avr2/Six5 pair pave the way to explore novel fungal virulence processes, and it will be valuable to explore whether similar combined effector functions exist in other plant-infecting microbes.

## Biometric pipeline to mine the effector literature

Louet et al. propose an innovative and efficient way to tackle literature covering seminal research on plant pathogen effectors. Their biometric pipeline, coined HIPEs (Highly Influential studies on plant Pathogen Effectors), selected 249 highly cited articles from the last two decades covering a wide variety of pathosystems. Countries, organizations, and journals were considered, along with the evolution of research trends, model molecules, and model organisms. HIPEs classified this dynamic scientific area into seven main Research Topics and 20 subtopics, with contrasting publication trends over time. Notably, HIPEs revealed that research on effectors from biotrophic and hemibiotrophic fungi is on the rise. Such biometric tools should be widely adopted by scientists to expertly mine the literature, identify trends and plan future research projects.

## Conclusion

The collection of articles published in the first instalment of this Research Topic (How can secretomics help unravel the secrets of plant-microbe interactions?) tackled *in vitro* secretomics, exosomes, proteases, effectors and bioinformatics tools, as well as crop breeding for microbial pathogen resistance. The second collection (Secretomics: More Secrets to Unravel on Plant-Fungus Interactions) highlighted the importance of extracellular vesicles as an alternative secretion pathway to facilitate the exchange of secreted components in a bidirectional flow between the host and its pathogen. This third volume unlocks more secrets and shows that gaining knowledge on the intricacies of plant-fungus associations demands efficient literature reviews, sophisticated experimental designs, cutting-edge technical platforms, powerful bioinformatics tools, as well as cross-disciplinary collaborations.

## Author contributions

DV wrote the first draft of the editorial. All corresponding authors edited the manuscript and approved the final version.

